# A report of a rare case of paratesticular spindle cell rhabdomyosarcoma in an adult patient

**DOI:** 10.1016/j.eucr.2023.102423

**Published:** 2023-05-03

**Authors:** Younesse Najioui, Nassira Karich, Anass Haloui, Aahd Belharti, Imane Kamaoui, Amal Bennani

**Affiliations:** a Pathology Department, Mohammed VI University Hospital, Oujda, Morocco; b Faculty of Medicine and Pharmacy, Mohammed I^st^ University, Oujda, Morocco; c Radiology Department, Mohammed VI University Hospital, Oujda, Morocco

**Keywords:** Spindle cell rhabdomyosarcoma, Embryonal rhabdomyosarcoma, Paratesticular region, Adult, Case report

## Abstract

Paratesticular embryonal rhabdomyosarcoma is a rare malignancy developed from a mesenchymal tissue of spermatic cord, testicular components. Spindle cell rhabdomyosarcoma (SCR) is a variant of embryonal rhabdomyosarcoma affecting the paratesticular region of adult patients and is even rarer. Given the limited guidelines available to manage SCR and the rarity of reported cases, our report aims to discuss a new case of this entity in a 66-years old Moroccan patient. Paratesticular SCR is a very rare tumor and requires attention from urologists to consider this entity as differential diagnosis when suspecting malignancies in the urogenital region.

## 1 Introduction

Paratesticular rhabdomyosarcoma is believed to originate from mesenchymal muscular tissues and occurs rarely in adult patients. It is more frequently diagnosed in children. The most common paratesticular rhabdomyosarcoma type is the embryonal subtype and spindle cell rhabdomyosarcoma (SCR) is one of its variants. Although it is known that SCR has a favorable prognosis in children, the available medical literature on prognosis in the adult population is less elucidated.^1^ In this case report, discuss a new case of a paratesticular SCR in a 66-year-old man. To the best of our knowledge, this is the first case to be reported in Morocco.

## 2 Case report

A 66-year-old man, with no particular medical history, presented a left scrotal mass evolving for six months. The scrotal mass was associated with pain on the left groin and weight loss. No difﬁculty with urination, dysuria, or hematuria were reported by the patient. Moreover, no history of recent trauma or sexually transmitted diseases were noticed during the patient interrogation. A clinical examination found a mobile 10 cm mass extending from the groin to the scrotum, with no associated hernia. The scrotal ultrasonography revealed a cystic mass of the spermatic cord. Both testicles had a normal echo-structure. Tumor markers including human beta chorionic gonadotropin βHCG), alpha-foeto-protein and prostate-specific antigen (PSA were of normal values. A computed tomography (CT) scan was indicated and did not reveal any positive lymph nodes or metastatic lesions.

**Fig. 1:**
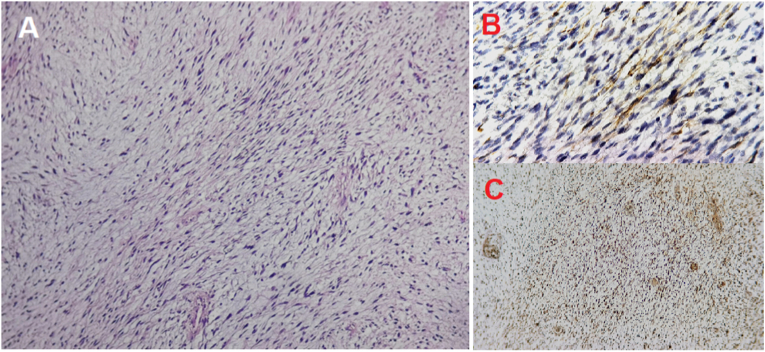
(A) Microphotography showing a sarcomatous proliferation made of fascicles of spindle cells. The tumor cells show moderate atypia with numerous mitotic figures. (B) Neoplastic cells with positive cytoplasmic expression of desmin. (C)Neoplastic cells with positive nuclear expression of Myo-D1.

**Fig. 2:**
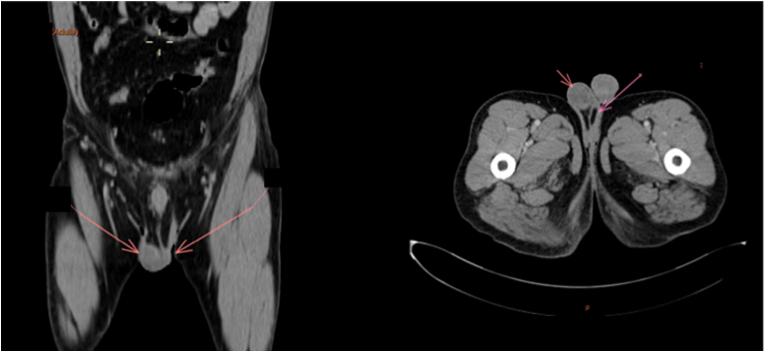
Follow up imaging showing no recurrence.

The patient underwent a surgical exploration that revealed the spermatic cord as an origin of the tumor. A conservative resection of the mass was then performed. The histopathological examination showed a proliferative lesion made of fascicles of spindle cells. These cells expressed desmin, myogenin and myo-D1 (Fig. 1). The other used antibodies during immunohistochemistry included smooth muscle actin antibody (SMA) and S-100 antibody and were both negative. A final pathological diagnosis of SCR was retained, and he benefited from six cycles of postoperative adjuvant chemotherapy using ifosfamide, doxorubicin and vincristine, given at 3-week intervals. Follow-up of our patient (Fig. 2) showed no local recurrence nor appearance of metastases during a period of 18 months.

## 3 Discussion

The paratesticular region is a rare location for neoplastic transformation. On the clinical level, a paratesticular tumor manifests as a rapidly growing intrascrotal mass. Pain is generally absent, although in our case a groin pain was reported by the patient, probably due to the important size of the tumor (10 cm). Paratesticular tumors are most commonly benign. Adenomatoid tumors and leiomyomas are the most frequent solid lesions along with epididymal cysts, spermatocele being the most frequent cystic lesions.^2^ Among all cases of paratesticular sarcomas in adult patients, rhabdomyosarcomas represent 7–10%.^2^ Most cases of paratesticular rhabdomyosarcomas have an embryonal histology. Among all variants of this subtype, the SCR is the rarest and it was ﬁrst documented in 1992 by Cavazzana in the adult population,^3^ as the case of our patient. Spindle cell variant of embryonal rhabdomyosarcomas accounts for 3% of all rhabdomyosarcomas and its occurrence in the paratesticular region is rarer but it stills the most common location for this tumor. Regarding imaging, ultrasonography is the standard initial diagnostic modality. It can reveal the lesion as a hypoechoic image and assess its vascularity on Doppler which appears increased. An associated hydrocele can also be revealed by ultrasonography.^1^ The histopathological macroscopic examination of SCR often shows a white whorled appearance with variable degree of necrosis and/or cystic degeneration.^3^ At the microscopic examination, SCR has a fascicular architecture, made of elongated spindle cell, containing a central nucleus. The cytoplasm is eosinophilic and may show signs of muscular differentiation in the form of rhabdomyoblasts. The differential diagnosis includes leiomyosarcoma and other spindle cell proliferations such as malignant peripheral nerve sheath tumor (MPNST), fibrosarcoma, solitary fibrous tumor, Triton tumor, which is an MPNST with rhabdomyoblastic differentiations, malignant fibrous histiocytoma, inflammatory myofibroblastic tumors, and low-grade myofibroblastic sarcoma (summarized in Table 1). An immunohistochemical study is always necessary for a definitive diagnosis and should show an expression of desmin, myogenin and myo-D by spindle cells and rhabdomyoblasts.^4^ On the genetic level, SCR is characterized by a distinctive MYOD 1 mutation.

**Table 1:** Different useful stains in the differential diagnosis between different paratesticular spindle cell proliferations according to the classification of the World Health Organization.

Spindle cell proliferations	MDM2, CDK4	Desmin	H-caldesmon	Ck	CD34	Myogenin	SMA
**Dedifferentiated liposarcoma**	+	+	+	rare	rare	–	–
**Leiomyoma, leiomyosarcoma**	–	+	+	–	–	–	–
**Rhabdomyoma, rhabdomyosarcoma**	–	+	+	–	–	+	–
**Fibroblastic/myofibroblastic tumors**	–	rare	–	–	+	–	–

Abbreviations: CD34: cluster of differentiation 34, CDK4: cyclin dependent kinase 4, Ck: cytokeratin, MDM2: mouse double minute 2, SMA: smooth muscle actin.

The prognosis of SCR in adults can be unfavorable if the diagnosis is delayed. In children for example, a delay in diagnosis was found associated with increased mortality and morbidity.^5^ Moreover, the presence of positive lymph nodes was more frequently marked in adults as compared to the pediatric population and is associated with reduced survival outcomes. There are no available guidelines for therapy decisions and the current therapeutic options are based on published case series and case reports and are similar to those available for the pediatric patients.^5^ Adjuvant chemotherapy after radical inguinal orchiectomy is offered and it is mainly based on the use of doxorubicin, actinomycin D, vincristine, and ifosfamide as well as other agents. In fact, the combination of these agents was demonstrated to improve survival outcome.

## 4 Conclusion

Paratesticular SCR is a very rare tumor and requires attention from urologists to consider this entity as differential diagnosis when suspecting malignancies for this urogenital region. The current optimal management combines radical orchiectomy followed by adjuvant chemotherapy. Overall, SCR has a favorable prognosis but studies to provide evidence for better management are awaited.

## Declaration of competing interest

None.
